# The E3 Ubiquitin Ligase TRIM21 Regulates Basal Levels of PDGFRβ

**DOI:** 10.3390/ijms24097782

**Published:** 2023-04-24

**Authors:** Niki Sarri, Natalia Papadopoulos, Johan Lennartsson, Carl-Henrik Heldin

**Affiliations:** 1 Department of Medical Biochemistry and Microbiology, Uppsala University, 751 23 Uppsala, Sweden; niki.sarri@imbim.uu.se (N.S.); natalia.papadopoulos@imbim.uu.se (N.P.); 2 Department of Pharmaceutical Biosciences, Uppsala University, 751 24 Uppsala, Sweden; johan.lennartsson@farmbio.uu.se

**Keywords:** TRIM21, ubiquitination, receptor tyrosine kinase, degradation

## Abstract

Activation of platelet-derived growth factor (PDGF) receptors α and β (PDGFRα and PDGFRβ) at the cell surface by binding of PDGF isoforms leads to internalization of receptors, which affects the amplitude and kinetics of signaling. Ubiquitination of PDGF receptors in response to ligand stimulation is mediated by the Casitas b-lineage lymphoma (Cbl) family of ubiquitin ligases, promoting internalization and serving as a sorting signal for vesicular trafficking of receptors. We report here that another E3 ligase, i.e., tripartite motif-containing protein 21 (TRIM21), contributes to the ubiquitination of PDGFRβ in human primary fibroblasts AG1523 and the osteosarcoma cell line U2OS and regulates basal levels of PDGFRβ. We found that siRNA-mediated depletion of TRIM21 led to decreased ubiquitination of PDGFRβ in response to PDGF-BB stimulation, while internalization from the cell surface and the rate of ligand-induced degradation of the receptor were not affected. Moreover, induction of TRIM21 decreased the levels of PDGFRβ in serum-starved cells, and even more in growing cells, in the absence of PDGF stimulation. Consistently, siRNA knockdown of TRIM21 caused accumulation of the total amount of PDGFRβ, both in the cytoplasm and on the cell surface, without affecting mRNA levels of the receptor. We conclude that TRIM21 acts post-translationally and maintains basal levels of PDGFRβ, thus suggesting that ubiquitination of PDGFRβ by TRIM21 may direct a portion of receptor for degradation in growing cells in a ligand-independent manner.

## 1. Introduction

The platelet-derived growth factor (PDGF) family comprises five disulfide-bonded dimers (AA, BB, CC, DD, AB) that act as mitogens and chemotactic agents for glial cells and cells of mesenchymal origin [1,2]. PDGF isoforms bind to α and β receptor tyrosine kinases (PDGFRα and PDGFRβ, respectively) with distinct affinities, causing receptor dimerization and auto-phosphorylation; this leads to the recruitment of downstream signaling molecules activating pathways that govern cell growth, cell proliferation and migration [1,2].

PDGF receptors are subject to ubiquitination, a post-translational modification that plays versatile roles in cellular events, e.g., in the regulation of protein stability and subcellular localization, DNA repair and cell proliferation [3,4]. Ligand-induced ubiquitination of PDGF receptors promotes their endocytosis and intracellular trafficking [5,6]. Members of the Cbl family of RING finger-containing E3 ligases are known to mediate the ubiquitination of tyrosine kinase receptors, including PDGFRβ [7,8,9].

Tripartite motif-containing 21 (TRIM21, Ro52) is a RING finger-containing E3 ligase, also known as cytosolic antibody receptor, belonging to the TRIM family of more than 80 members. TRIM21 contains N-terminal RING and B-box domains, a central coiled-coil domain and a carboxyl-terminal PRY/SPRY domain [10]. The RING domain is critical for the interaction with E2 enzymes and catalyzes the transfer of ubiquitin to protein substrates. The E3 ligase activity of TRIM21 is constitutively repressed by its B-box domain that can be activated by phosphorylation of Ser in the LxxIS motif of the RING domain [11]. The PRY/SPRY domain is essential for protein–protein interactions and subcellular localization [12] and binds to the Fc region of IgG molecules with high affinity [13]. This feature of TRIM21 has been explored to design a selective protein targeting strategy “Trim-Away” for acute and rapid degradation of endogenous proteins [14].

TRIM21 has an important role in immune host defense, signal transduction and cell cycle regulation [10], and has also been implicated in tumorigenesis [15]. Moreover, TRIM family proteins have emerged as key players in the regulation of protein quality control, which, through ubiquitination, can effectively direct misfolded proteins or protein aggregates for degradation by the ubiquitin proteasomal pathway (UPS) [16]. TRIM21 binds to and polyubiquitinates human IgG1 heavy chain and interacts with the molecular chaperone p97/VCP, mediating quality control of IgG1 heavy chain through the ER-associated degradation (ERAD) system [17]. Furthermore, TRIM21 targets aggregates of misfolded tau, a cytoplasmic protein that accumulates in patients with Alzheimer’s disease, for degradation in proteasomes [18]. Additionally, the family of tripartite motif proteins (TRIM) has been reported as regulators of selective autophagy, either serving as scaffold proteins or acting via ubiquitin-dependent mechanisms [19]. TRIM21 directly interacts with and ubiquitinates SQSTM1/p62, a ubiquitin-binding scaffold protein, that acts as a receptor of autophagic cargo [20].

In this study, we show that TRIM21 promotes ubiquitination of PDGFRβ. While it does not appreciably affect the stability or the cell surface clearance of PDGFRβ upon ligand stimulation, TRIM21 decreases the basal levels of PDGFRβ, presumably by committing a fraction of synthesized and stored receptor for degradation.

## 2. Results

### 2.1. Depletion of TRIM21 Reduces PDGF-BB-Induced PDGFRβ Ubiquitination

**Figure 1:**
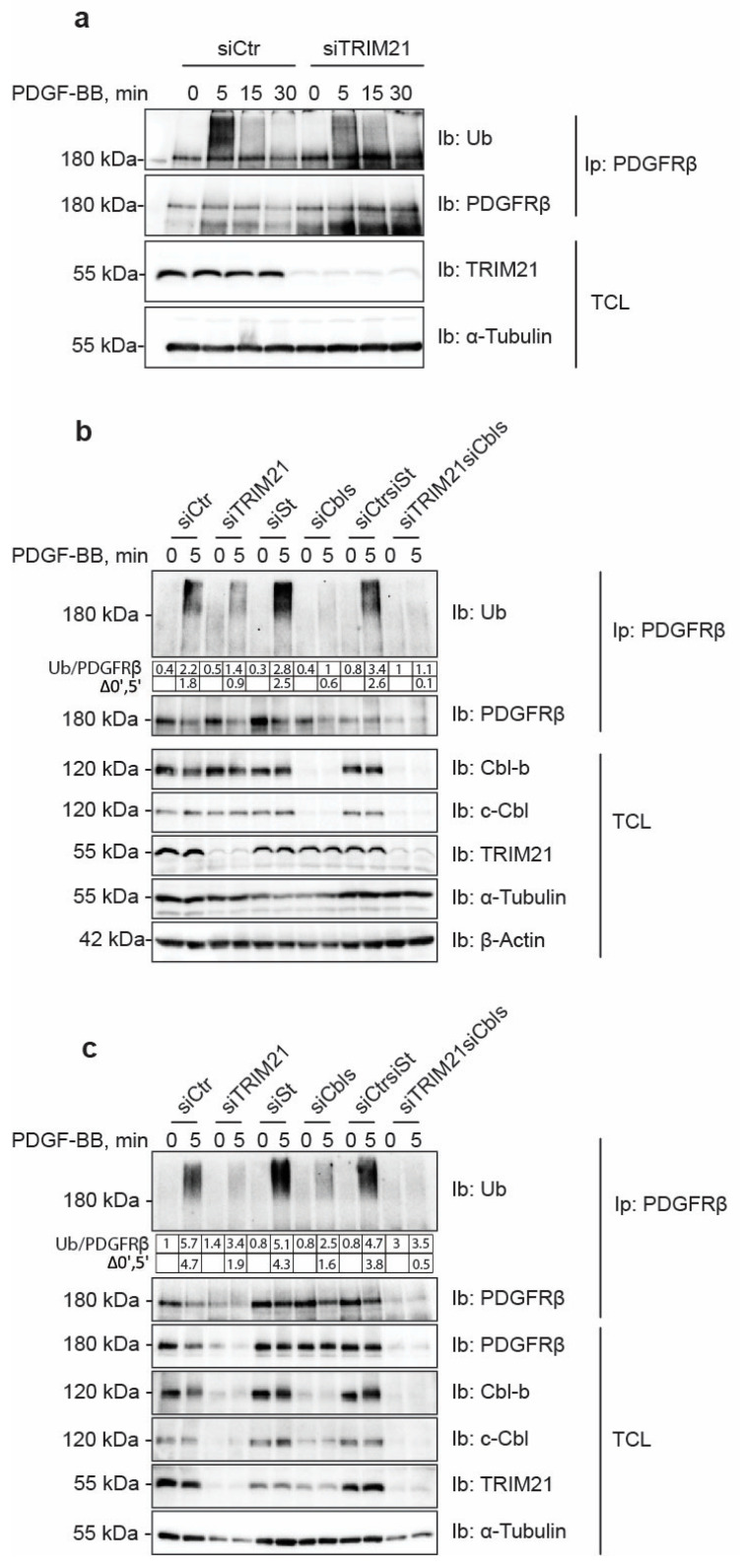
TRIM21 is an E3 ubiquitin ligase of PDGFRβ. (**a**) SiRNA-mediated depletion of TRIM21 decreases the ubiquitination of PDGFRβ. After transient silencing of TRIM21 and serum-starvation, U2OS cells were stimulated with PDGF-BB for the indicated time periods, followed by PDGFRβ immunoprecipitation with anti-PDGFRβ antibody (CTβ). Immunoprecipitation eluates were blotted for Ubiquitin (Ub) and PDGFRβ. Total cell lysates (TCL) were blotted for TRIM21 and tubulin. The experiment was repeated three times. (**b**,**c**) Simultaneous knock-down of TRIM21, c-Cbl and Cbl-b completely removes the ubiquitination of PDGFRβ. The experiment was performed in U2OS (**b**) and in AG01523 (**c**) cells following the protocol described in (**a**). The level of ubiquitination was quantified and divided by the level of immunoprecipitated receptor; the values are included as Ub/PDGFRβ under the blot. In order to estimate the amount of ubiquitination, the values for non-ubiquitinated receptor at 0 min were subtracted from 5 min of stimulation and the difference was presented as “∆0′,5′”. “siCtr” indicates siRNA control from the same manufacturer as TRIM21 siRNA (Origene); “siSt” indicates siRNA control for Cbl-b and c-Cbls siRNAs (“siCbls”) from ThermoScientific; for triple knockdowns, control siRNAs were mixed together and abbreviated as “siCrtsiSt. TCL was immunoblotted for Cbl-b, c-Cbl, TRIM21, α-tubulin and β-actin. The experiments presented in (**b**,**c**) were repeated two times in each of the cell lines.

Members of the Cbl family of E3 ubiquitin ligases are known to promote the ubiquitination of PDGFRβ. We have previously shown that silencing of c-Cbl and Cbl-b in AG01523 human fibroblasts by siRNA efficiently removed most of the ubiquitination of PDGFRβ, while some residual ubiquitination was left. We identified TRIM21 in a mass spectrometry screen for PDGFRβ interactors. In order to explore whether TRIM21 can act as an E3 ligase for PDGFRβ, we depleted human osteosarcoma cells (U2OS) of TRIM21 using siRNA. Knockdown of TRIM21 partially decreased the ubiquitination of PDGFRβ when cells were starved overnight, followed by stimulation with PDGF-BB (most notable after 5 min of stimulation; Figure 1a). In order to assess a possible complementary role of TRIM21 and members of the Cbl family on PDGFRβ ubiquitination, we performed simultaneous depletion of TRIM21, c-Cbl and Cbl-b; we observed that the triple knockdown of these E3 ligases completely prevented the ubiquitination of PDGFRβ in U2OS cells (Figure 1b) and in AG01523 fibroblasts that were used to validate the results obtained for the U2OS cell line (Figure 1c). Treatment of AG1523 cells with siTRIM21 caused loss of cells and thus lower amounts of protein and immunoprecipitated receptor; this does not change the conclusion from the experiment. These findings indicate that c-Cbl, Cbl-b and TRIM21 are all capable of ubiquitinating PDGFRβ.

### 2.2. Induced Expression of TRIM21 Affects the Signaling Events Downstream of PDGFRβ 

**Figure 2:**
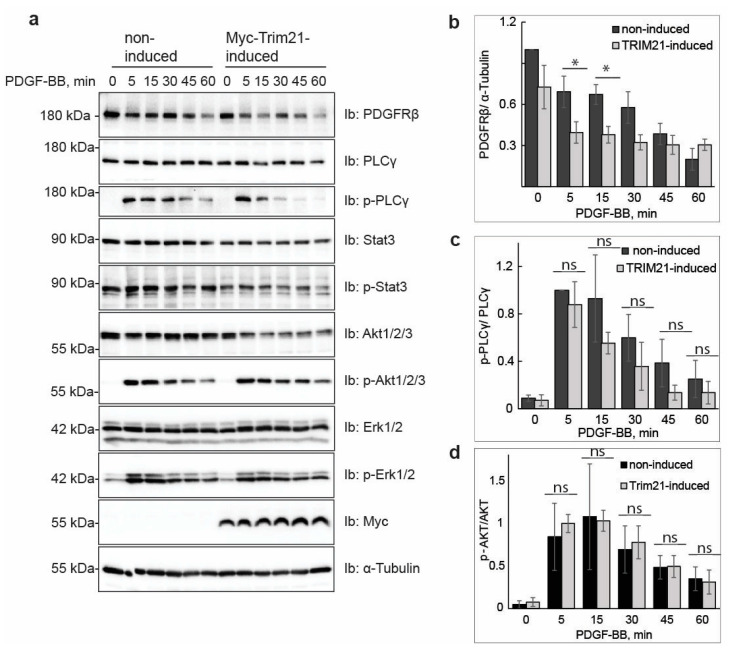
TRIM21 induction in tet-inducible U2OS cell line results in decreased levels of PDGFRβ and affects activation of PLCγ. After induction of TRIM21 with doxycycline and overnight serum-starvation, the cells were stimulated with PDGF-BB for the indicated time periods. Total cell lysates (TCL) were prepared and subjected to SDS-PAGE. Immunoblotting (Ib) was performed with antibodies against PDGFRβ, PLCγ, pY783 PLCγ, Stat3, pStat3 (Y705), Akt1/2/3, pS473 Akt1/2/3, Erk1/2, pThr202/pThr204 Erk1/2, Myc and α-tubulin (**a**). Quantification of four independent experiments is presented for PDGFRβ (**b**), p-PLCγ (**c**) and p-Akt (**d**). * *p* < 0.05; “ns”—non-significant.

We investigated the impact of TRIM21-induced ubiquitination of PDGFRβ on the subsequent signaling events activated upon stimulation of serum-starved U2OS cells with PDGF-BB (Figure 2a). We found that induction of TRIM21 significantly decreased the levels of PDGFRβ (Figure 2a,b). Moreover, activation of phospholipase Cγ (PLCγ) was somewhat decreased upon induction of TRIM21, but not significantly (Figure 2a,c), while other signaling effectors, such as Akt (Figure 2a,d), STAT3 and ERK1/2 MAP-kinase (Figure 2a), were not affected. Thus, the decreased basal levels of PDGFRβ after induction of TRIM21 may explain the decrease in activation of certain signaling molecules, such as PLCγ, whereas the decrease in receptor levels was not sufficient to affect other signaling pathways.

### 2.3. TRIM21 Affects the Total Amounts of PDGFRβ at the Cell Surface

**Figure 3:**
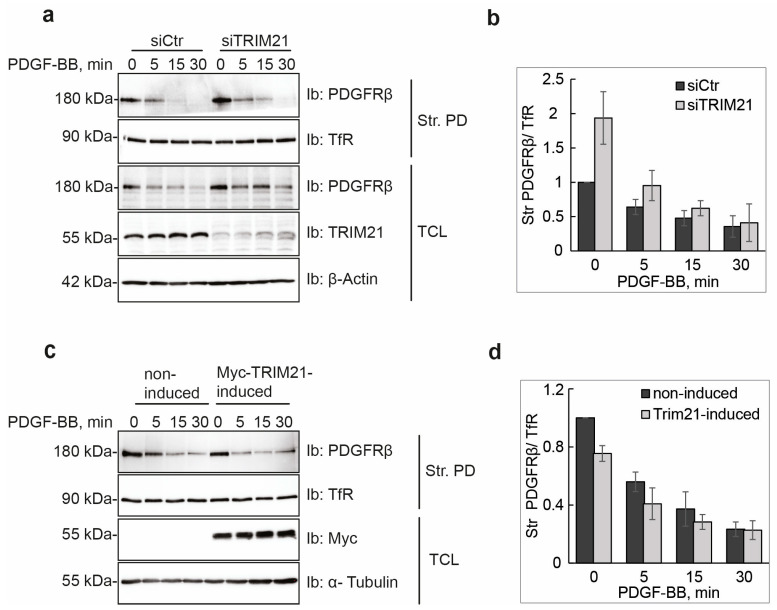
TRIM21 affects the total levels of PDGFRβ at the cell surface. (**a**,**b**) Total levels of PDGFRβ are increased on the cell surface after depletion of TRIM21. TRIM21 was transiently knocked-down in U2OS cells and cells were serum-starved overnight. After stimulation for the indicated periods of time with PDGF-BB, cell surface proteins were biotinylated and cells were lysed and incubated with streptavidin (ST)-agarose (Str. PD; streptavidin pulldown) and immunoblotted (Ib) for PDGFRβ and transferrin receptor (TfR). Total cell lysates (TCL) were immunoblotted for PDGFRβ, TRIM21 and α-tubulin. Quantification of three experiments is presented in (**b**). (**c**,**d**) Induction of TRIM21 in tet-inducible U2OS-TRIM21 cell line downregulates total levels of PDGFRβ on the cell surface (**c**). The experiment was performed using the protocol described in (**a**). Quantification of four experiments is presented in (**d**).

Ubiquitination has been linked to internalization and intracellular sorting of receptors [5]. We next determined if TRIM21 regulates subcellular trafficking of PDGFRβ and internalization from the cell surface in response to ligand stimulation. To this end, we labeled cell surface PDGFRβ in U2OS cells with biotin and pulled down the biotinylated receptor that was left on the cell surface after different periods of stimulation with PDGF-BB, using streptavidin agarose beads. Interestingly, we found that depletion of TRIM21 led to an increased total level of PDGFRβ compared to control conditions (Figure 3a). Moreover, the amount of PDGFRβ at the cell surface in unstimulated, TRIM21-depleted cells was higher compared to the basal levels of PDGFRβ in control cells (Figure 3b), which was reproducible but did not reach significance due to variation of the levels between triplicates. Consistently, we observed a decrease up to approximately 20% in the basal levels of PDGFRβ at the cell surface upon the induction of Myc-TRIM21 (Figure 3c,d). However, TRIM21 neither affected ligand-induced PDGFRβ internalization from the cell surface in TRIM21-depleted nor in Myc-TRIM21-induced U2OS cells.

### 2.4. TRIM21 Controls Basal Levels of PDGFRβ in Growing Cells, but Not PDGFRβ mRNA Levels or the Stability of the Receptor during Ligand-Mediated Degradation

**Figure 4:**
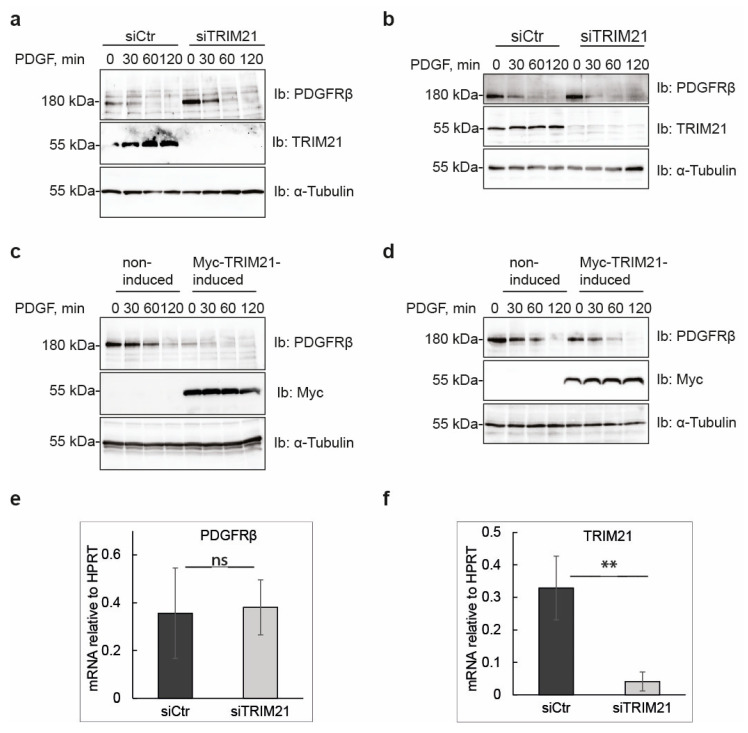
TRIM21 controls basal levels of PDGFRβ in growing cells but does not affect receptor mRNA levels. (**a**,**b**) Depletion of TRIM21 increases basal levels of PDGFRβ but not the rate of degradation after stimulation with PDGF-BB. After transient silencing of TRIM21 in fully growing (**a**) or serum-starved cells for 16 h (**b**), U2OS cells were pretreated with cycloheximide (CHX) for 1 h and stimulated with PDGF-BB for the indicated time periods. Expression levels of PDGFRβ, TRIM21 and α-tubulin, as a loading control, were determined by immunoblotting (Ib) of total cell lysates (TCL) (**c**,**d**). Overexpression of TRIM21 decreases the total levels of PDGFRβ but does not affect its stability of upon ligand stimulation. The experiment was performed using the tet-inducible U2OS-TRIM21 cell line, that were growing in full media (**c**) or serum-starved for 16 h (**d**), treated with cycloheximide and stimulated with PDGF-BB as described in (**a**). TCL was subjected to immunoblotting for PDGFRβ, MYC-epitope to detect MYC-tagged TRIM21 and α-tubulin. (**e**,**f**) mRNA expression levels of PDGFRβ do not change upon knockdown of TRIM21. Total RNA was extracted from U2OS cells that were treated with control or TRIM21 siRNA for 72 h. mRNA was reverse transcribed and amplified to detect expression levels of *PDGFRβ* (**e**) or *TRIM21* (**f**). Expression was normalized against the control gene *HPRT*, the average values for four independent repeats are presented in the graph. ** *p* < 0.01; “ns”—non-significant.

Since TRIM21 expression affected the total levels of PDGFRβ, we determined the levels and stability of the mature receptor in both serum-starved cells and in cells growing in full media. When U2OS cells were pre-treated with cycloheximide to block new protein synthesis and stimulated with PDGF-BB to promote ligand-induced receptor degradation, we observed that depletion of TRIM21 led to a larger increase of total PDGFRβ levels in actively growing cells (Figure 4a) compared to serum-starved cells (Figure 4b), while induction of TRIM21 led to stronger downregulation of PDGFRβ protein in actively growing cells (Figure 4c) than in serum-starved cells (Figure 4d). Ligand-induced degradation of mature PDGFRβ protein was not affected by TRIM21 expression levels in either of the cases. 

In order to exclude the possibility that TRIM21, in addition to ubiquitinating PDGFRβ, affects its levels indirectly by affecting transcription of PDGFRβ, we analyzed mRNA levels of PDGFRβ upon siRNA knockdown of TRIM21 (Figure 4e); the efficiency of the TRIM21 knockdown is shown in Figure 4f. We found that the mRNA expression levels of *PDGFRβ* were not changed upon depletion of TRIM21, thus indicating that TRIM21 controls PDGFRβ amount post-translationally.

### 2.5. PDGFRβ Accumulates in the Cytoplasm and by the Plasma Membrane upon Knockdown of TRIM21, While Being Degraded upon Induction of the E3 Ligase

**Figure 5:**
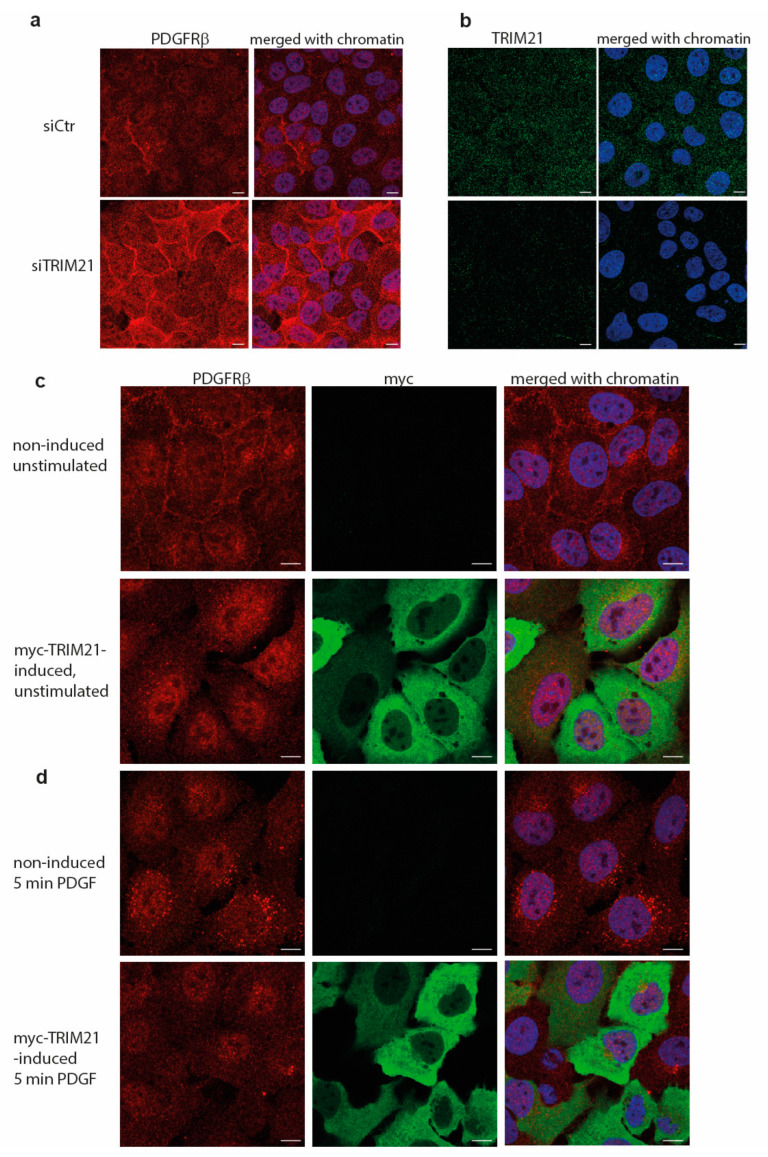
(**a**) Immunofluorescence staining of PDGFRβ in U2OS cells that were treated with control siRNA (“siCtr”) or depleted of TRIM21 protein (“siTRIM21”). Single channel staining of PDGFRβ is presented (red) and merged with nuclear staining with DAPI (blue). (**b**) Immunofluorescence staining of endogenous TRIM21 (green) shows weak cytoplasmic distribution in control cells (“siCtr”) and efficient knockdown in (siTRIM21) cells; staining merged with nuclei (blue) images are also presented. Confocal images in (**a**,**b**) were taken with 63× oil objective and zoom 0.5. (**c**,**d**) Immunofluorescence staining of PDGFRβ in myc-TRIM21-inducible U2OS cells cultured in 10% FBS after induction of TRIM21 before (**c**) or after (**d**) stimulation with PDGF-BB for 5 min. PDGFRβ staining is in the red; TRIM21 (myc) staining is in green; chromatin counterstained with DAPI is in the blue. Merged images for all three channels are presented on the right. Confocal images in (**c**,**d**) were taken with 63× oil objective and zoom 0.7. Scale bars are 10 µm.

In an attempt to map the effect of TRIM21 on the subcellular localization of PDGFRβ, we performed immunostaining of the receptor upon depletion (Figure 5a,b) or during induction (Figure 5c) of TRIM21 in growing U2OS cells. We observed that the staining for PDGFRβ was accumulated in the cytoplasm and, in particular, by the plasma membrane in cells depleted of TRIM21 (Figure 5a); the efficiency of knockdown was confirmed by immunostaining (Figure 5b), as well as immunoblotting. PDGFR levels at the plasma membrane and in the cytoplasm decreased in actively growing cells when TRIM21 was induced (Figure 5c). Additionally, when cells were stimulated with PDGF-BB for 5 min, fewer PDGFRβ clusters were observed in the cells expressing TRIM21 (Figure 5d), which is consistent with the results presented in Figure 2, where the level of PDGFRβ in TRIM21-expressing cells was shown to be decreased. 

## 3. Discussion

The family of Cbl E3 ligases has been reported to play a key role in the ubiquitin-mediated internalization of PDGFRβ [21]. We found that depletion of c-Cbl and Cbl-b robustly decreased the ubiquitination of PDGFRβ, albeit leaving residual ubiquitination of the receptor, particularly in AG1523 fibroblasts. In this study, we identified TRIM21 as an additional E3 ligase for PDGFRβ. Depletion of TRIM21 affected the ubiquitination status of PDGFRβ in both AG1523 fibroblasts and U2OS cells and removed the residual ubiquitination of PDGFRβ in AG1523 cells that remained after knockdown of Cbl family members. This suggests that TRIM21 ubiquitinates the receptor and that it may complement the action of Cbls, possibly in a cell type-specific and context-dependent manner. 

Manipulation of TRIM21 levels in U2OS cells did not affect ligand-mediated internalization from the cell surface or ligand-mediated degradation of PDGFRβ, while it consistently impacted on the total levels of receptor in unstimulated cells. This suggests that TRIM21 is involved in the regulation of basal PDGFRβ protein levels in a ligand-independent fashion. Interestingly, we observed that the effect of TRIM21 depletion or overexpression on the levels of PDGFRβ was more profound when cells were growing in 10% FBS than in starved cells. This may be explained by the fact that there is more protein synthesis and protein turnover in actively growing cells than in cells that were serum starved.

We found that depletion of TRIM21 levels did not affect mRNA levels of PDGFRβ, thus indicating that TRIM21 works on the receptor protein post-translationally. TRIMs have been known to participate in the clearance of misfolded proteins through distinct mechanisms including ubiquitin-proteasome pathway, autophagy and ER-associated degradation (ERAD) [16]. Specifically, TRIM21 has been reported to direct misfolded proteins from the endoplasmic reticulum (ER) for proteasomal degradation [17]. Therefore, it would be interesting to evaluate the possibility that the action of TRIM21 may be coupled to the new synthesis of PDGFRβ. It is also possible that TRIM21 does not have a housekeeping protein quality control function for PDGFRβ, but rather may be induced under special conditions that lead to activation or upregulation of TRIM21. This possibility is consistent with the findings that TRIM21 is an interferon-inducible gene, which is produced in cells in an autoinhibited conformation and can be activated by IKKβ and TBK1 via serine phosphorylation [11]. 

PDGFRβ has been reported to be degraded both by proteasomal [22] and lysosomal [4] pathways. The majority of studies have focused on ligand-mediated degradation, and it is currently unknown whether there is a specific degradative pathway that controls the total levels of newly synthesized PDGFRβ, or affects its levels during maturation, storage and/or delivery to the plasma membrane. Our findings support the notion that TRIM21-dependent ubiquitination regulates the total pool of PDGFRβ under basal conditions. This ligand-independent regulatory pathway, consequently, may control the number of PDGFRβ molecules at the plasma membrane, which may influence the PDGF-BB-induced activation of PLCγ and other signaling pathways. In addition to this, TRIM21 may associate with the receptor at the plasma membrane and impact the ubiquitination during ligand stimulation, as we found that ubiquitination levels of receptor were decreased in cells depleted of TRIM21. 

Interestingly, TRIM21 has been found to directly bind to its cargo and assemble autophagic machinery to execute degradation [23]. A possibility that remains to be elucidated is that TRIM21 specifically targets misfolded or excessive PDGFRβ protein for autophagic turnover.

In conclusion, we present evidence for a TRIM21-regulated mechanism to control basal PDGFRβ levels, complementing the ligand-induced PDGFRβ ubiquitination by Cbl family members. The precise mechanism of where in the cell TRIM21 ubiquitinates the receptor and directs it to degradation remains to be elucidated.

## 4. Materials and Methods

### 4.1. Reagents and Antibodies

Primary antibodies against β-actin (AC-15, #A5441) and α-tubulin (B-5-1-2, #T6074) were purchased from Sigma-Aldrich (St. Lous, MO, USA), and antibodies against ubiquitin from either Santa Cruz Biotechnology (Dallas, TX, USA) (sc-8017) or Invitrogen (Waltham, MA, USA) (#16-6078-82). Rabbit polyclonal antibodies recognizing PDGFRβ (CTβ) were homemade [24]; goat polyclonal anti-PDGFRβ antibodies were from R&D (Minneapoilis, MN, USA) (AF385). Primary antibodies against Cbl-b (D3C12, #9498), c-Cbl (#2747), GAPDH (D16H11, #5174), Akt (#9272S), phosphorylated Akt (pSer473, D9E, #4060/pThr308, 244F9, #4056), p44/p42 MAPK (Erk1/2, 137F5, #4695), phosphorylated p44/p42 Erk1/2 MAPK (pThr202/pThr204, #9101), PLCγ1 (#2822), phosphorylated PLCγ1 (pTyr783, #2821), STAT3 (79D7, #4904), phosphorylated STAT3 (pTyr705, D3A7, #9145) and LC3B (#3868) were purchased from Cell Signaling Technology (Danvers, MA, USA). Primary antibodies against TRIM21 that were used for immunoblotting were from Santa Cruz (sc25351) and for immunostaining from Abcam (Cambridge, UK) (ab91423); myc antibodies were from Cell Signaling (9811). Primary antibodies against the transferrin receptor (ab84036) were obtained from Abcam. Secondary antibodies for immunoblotting were HRP-conjugated anti-mouse IgG (#62–6520) and HRP-conjugated anti-rabbit IgG (#65–6120) from Invitrogen. Fluorescent secondary antibodies were anti–mouse IgG–Alexa Fluor 488 (green), anti–rabbit IgG–Alexa Fluor 546 (red) and anti–rabbit Alexa Fluor 633 (far red) from ThermoScientific (Waltham, MA, USA). DAPI was purchased from ThermoScientific, puromycin from Invitrogen and doxycycline from Takara Bio (Kusatsu, Japan).

### 4.2. Cell Culture and Treatments

The human osteosarcoma cell line U2OS (Uppsala University, Uppsala, Sweden) was cultured in Dulbecco’s Modified Eagle’s medium (DMEM) (Sigma-Aldrich), supplemented with 10% fetal bovine serum FBS (Biowest, Nuaille, France) at 37 °C in 5% CO_2_ humidified atmosphere. The U2OS doxycycline-inducible cell lines (see below) were cultured in DMEM, supplemented with 2.0 μg/mL puromycin and 10% FBS. Starvation media was DMEM, supplemented with 0.1% FBS. Cell monolayers were stimulated with 20 ng/mL PDGF-BB (Chiron Corp., Emeryville, CA, USA) for biochemical experiments. For stability experiments, cells were pretreated with 50 µg/mL cycloheximide for one hour. For the induction of TRIM21, the tet-inducible U2OS cell line was pretreated with 100 ng/mL doxycycline from 24 to 48 h. Human foreskin fibroblasts AG01523 (Coriell Cell Repositories, Camden, NJ, USA) were cultured in Eagle’s minimum essential medium, supplemented with 2 mM L-glutamine and 10% FBS. For starvation, cells were incubated in the same medium containing 0.1% FBS. Last, for autophagic induction, cells were maintained in Earle’s balanced salts solution (EBSS) (ThermoScientific, Waltham, MA, USA) for five hours at 37 °C in 5% CO_2_ humidified atmosphere.

### 4.3. Generation of Tet-Inducible Cell Lines

Lenti-X Tet-One Inducible Expression System (Takara Bio, Kusatsu, Japan) was used for the generation of tet-inducible cell lines. Myc-TRIM21 was cloned into the pLVX-TetOne vector, using In-Fusion HD Cloning Kit (Takara Bio, Kusatsu, Japan) and the construct was tested by transient transfection. Viral particles were produced from the Lenti-X vectors using Lenti-X Packaging Single Shots (VSV-G) and transfected to the 293T cells cultured in 10-cm dishes in 8 mL DMEM supplemented with 10% FBS to obtain enough lentivirus. Cell culture media containing virus particles was collected after 24 h and after 48 h post-transfection, mixed, diluted trice in cell culture medium, supplemented with 8 μg/mL polybrene and added to U2OS cells cultured in 6-well plates. Cell culture medium was changed after 16 h to DMEM supplemented with Tet System Approved FBS (Takara Bio, Kusatsu, Japan) and puromycin (2.0 μg/mL). Surviving cells were cultured in media with puromycin. 

### 4.4. siRNA Transfection

U2OS cells were transiently transfected with 20 nM siRNA of trilencer-27 TRIM21 siRNA (#SR304594) or 20 nM scrambled negative control siRNA (#SR30004; both from OriGene, Rockville, MD, USA) using SilentFect (BioRad Laboratories, Inc., Hercules, CA, USA) for 28 h at 37 °C, according to the manufacturer’s protocol. Down-regulation of Cbl-b and c-Cbl was performed by using 20 nM siRNA CBLBHSS101420 and 40 nM siRNA CBLHSS101418 from Invitrogen, respectively. Stealth RNAi Negative Control Duplex (12935-112) from Invitrogen was used as a control. Transfection of siRNA was conducted for 28 h with SiLentFect from Bio-Rad (Hercules, CA, USA), and experiments were performed after an additional 20 h. Levels of knockdown were tested by immunoblotting.

### 4.5. Immunoprecipitation and Immunoblotting

After starvation and stimulation of 50% confluent cell monolayers with PDGF-BB (20 ng/mL) for the indicated time periods, cells were washed once in ice-cold phosphate-buffered saline (PBS) and lysed in RIPA lysis buffer (1% Triton X-100, 0.5% deoxycholate, 0.1% sodium dodecylsulfate (SDS), 10% glycerol, 20 mM Tris, pH 7.4, 150 mM NaCl), supplemented with 1 mM Pefa Block and 1 mM sodium orthovanadate for 15 min on ice. The cell lysates were centrifuged at 13,000 rpm for 15 min at 4 °C and incubated with the primary antibodies overnight at 4 °C with end-over-end rotation, followed by one-hour incubation with protein A/G magnetic beads (ThermoScientific). The beads were washed three times with ice-cold lysis buffer and the adsorbed proteins were eluted in 1% SDS sample buffer by heating at 95 °C for five min. The protein samples were subjected into SDS–polyacrylamide gel electrophoresis (SDS-PAGE), and electro-transferred to PVDF membranes (Immobilon); the membranes were blocked in 5% bovine serum albumin (BSA) in PBS, 0.1% Tween-20 and incubated at 4 °C overnight with primary antibodies. After three washes in PBS, 0.1% Tween-20, the membranes were incubated with horseradish peroxidase-conjugated secondary antibodies for one hour at room temperature. The proteins were visualized with the enhanced chemiluminescence (ECL) detection system on a charge-coupled device (CCD) camera (BioRad) and quantified using Bio-Rad ImageLab 6.0.1 software (Hercules, CA, USA).

### 4.6. Cell Surface Biotinylation Assay

Following stimulation with 20 ng/mL PDGF-BB at the indicated periods of time, cells were incubated with 0.3 mg/mL Sulfo-NHS-SS-Biotin (Pierce, Waltham, MA, USA) in PBS for one hour at 4 °C to label cell surface proteins. Excess biotin was then quenched with 50 mM Tris (pH 8.8) and cells were washed once in ice-cold PBS. Cells were lysed in RIPA buffer and lysates were incubated with streptavidin-sepharose (GE Healthcare, Chicago, IL, USA) for one hour at 4 °C. Protein-bound streptavidin beads were washed three times in lysis buffer, and biotinylated proteins were eluted by addition of SDS sample buffer and subjected to SDS-PAGE, followed by immunoblotting.

### 4.7. mRNA Extraction and Quantitative PCR

Total RNA from U2OS cells that were transiently transfected with control or TRIM21 siRNAs (as described above) was extracted 72 h post-transfection using NucleoSPin RNA Plus kit (Macherey-Nagel, Duren, Germany). One µg of total RNA was used to produce cDNA using High Capacity cDNA Reverse transcription kit (#10400745, Applied Biosystems, Waltham, MA, USA). cDNA was diluted 15 times and subjected to quantitative PCR (qPCR), using qPCRBio reagent and the following primers: HPRT forward 5′-CCTGGC GTCGTGATTAGTGAT-3′, HPRT reverse 5′-AGACGTTCAGTCCTG TCCATAA-3′; PDGFRβ forward 5′-AGCACACTGCGTCTGCAGCA-3′; and PDGFRβ reverse 5′-TGA GCACCACCAGGGCCAG-3′; TRIM21 forward 5′-GTCCTGGAAAGGAGTGAGTCC, TRIM21 reverse 5′-CTGAAAGTATCAGCCACGGATT. mRNA expression levels for PDGFRβ and TRIM21 were normalized against HPRT control gene. 

### 4.8. Immunofluorescence

U2OS cells were grown on coverslips in full media and induced for expression of TRIM21 (tet-inducible cell lines) or depleted for TRIM21 with siRNA as described above. Cells were fixed in 3.7% paraformaldehyde for 10 min, washed twice in PBS, permeabilized in 0.1% SDS for 10 min, blocked in 1% BSA/PBS solution for 1 h and primary antibodies were applied overnight. Slides were washed 5 times in PBS, incubated with fluorescent secondary antibodies for 1 h, washed 5 times with PBS, counterstained with DAPI for 5 min, mounted in Vectashield mounting media (Vector Labs, Newark, CA, USA) and analyzed by scanning confocal microscopy. Images were acquired using Zeiss LCM700 inverted confocal microscope with numerical aperture 1.4 oil objectives at 1128 × 1128 pixels. Zen (black edition) software (Zeiss, Oberkochen, Germany) and a high-resolution AxioCam microscope camera (ZEISS) were used at the Biological Visualization Facility (BioVis, Uppsala University). Images were exported as merged TIFF files with 8-bit resolution and individual channels were separated in Photoshop; brightness was adjusted equally on all images within each experiment. 

### 4.9. Statistical Analysis

Statistical analyses were carried out using Microsoft Excel. The statistical significance of differences among mean values was calculated based on at least three individual repeats by two-tailed *t* test with unequal variance. All experiments were performed three times unless indicated otherwise in the figure legend.

## Data Availability

Data supporting reported results can be obtained from the authors.
